# The prevalence and locations of bone metastases using whole-body MRI in treatment-naïve intermediate- and high-risk prostate cancer

**DOI:** 10.1007/s00330-020-07363-x

**Published:** 2020-11-03

**Authors:** Fredrik Ottosson, Eduard Baco, Peter M. Lauritzen, Erik Rud

**Affiliations:** 1 Department of Urology, Oslo University Hospital, Aker, Oslo, Norway; 2 Division of Radiology and Nuclear Medicine, Oslo University Hospital, Aker, Oslo, Norway; 3 Division of Radiology and Nuclear Medicine, Oslo University Hospital, Aker, Postboks 4959, Nydalen, 0424 Oslo, Norway

**Keywords:** Prostatic neoplasm, Neoplasms metastases, Magnetic resonance imaging, Risk assessment

## Abstract

**Objective:**

The aim of this study was to assess the prevalence and distribution of bone metastases in treatment-naïve prostate cancer patients eligible for a metastatic workup using whole-body MRI, and to evaluate the results in light of current guidelines.

**Methods:**

This single-institution, retrospective study included all patients with treatment-naïve prostate cancer referred to whole-body MRI during 2016 and 2017. All were eligible for a metastatic workup according to the guidelines: PSA > 20 ng/ml and/or Gleason grade group ≥ 3 and/or cT ≥ 2c and/or bone symptoms. The definition of a metastasis was descriptive and based on the original MRI reports. The anatomical location of metastases was registered.

**Results:**

We included 161 patients with newly diagnosed prostate cancer of which 36 (22%) were intermediate-risk and 125 (78%) were high-risk. The median age and PSA were 71 years (IQR 64–76) and 13 ng/ml (IQR 8–28), respectively. Bone metastases were found in 12 patients (7%, 95% CI: 4–13), and all were high-risk with Gleason grade group ≥ 4. The pelvis was affected in 4 patients, and the spine + pelvis in the remaining 8. No patients demonstrated metastases to the spine without concomitant metastases in the pelvis. Limitations are the small number of metastases and retrospective design.

**Conclusion:**

This study suggests that the overall prevalence of bone metastases using the current guidelines for screening is quite low. No metastases were seen in the case of Gleason grade group ≤ 3, and further studies should investigate if it necessary to screen non-high-risk patients.

**Key Points:**

*• The overall prevalence of bone metastases was 7% in the case of newly diagnosed intermediate- and high-risk prostate cancer.*

*• The prevalence in high-risk patients was 10%, and no metastases were seen in patients with Gleason grade group ≤ 3.*

*• The pelvic skeleton is the main site, and no metastases occurred in the spine without concomitant pelvic metastases.*

## Introduction

Identification of bone metastases in patients with prostate cancer is of great importance in order to choose the appropriate treatment. The European Association of Urology (EAU) currently recommends a metastatic workup in patients with prostate-specific antigen (PSA) > 20 ng/ml, and/or Gleason score ≥ 7b corresponding to the International Society of Urogenital Pathology (ISUP) Gleason grade group ≥ 3 and/or cT ≥ 2c and/or bone symptoms [1]. The new criterion, compared with the guidelines prior to 2015, is the recommendation to examine all patients with Gleason grade group 3, regardless of PSA or clinical T-stage [1, 2].

The most common methods available for detection of bone metastases are magnetic resonance imaging (MRI), bone scan (BS), and choline positron emission tomography (PET) CT. BS is currently recommended by the EAU, although MRI is regarded to be the most accurate method, with a sensitivity and specificity of around 90–100% [3–5]. The American Society of Clinical Oncology recommends whole-body MRI as an alternative or supplement to conventional BS or CT [6].

The pattern of metastatic spread of prostate cancer is not entirely understood, but it is considered to be an “ascending disease” [7, 8]. This refers to an initial local spread in the pelvis, before ascending in the axial skeleton and lymph nodes. For this reason, the pelvis is the most prevalent site of bone metastases [9, 10]. The aim of this study was to assess the prevalence and distribution of bone metastases using whole-body MRI in treatment-naïve prostate cancer patients eligible for a metastatic workup according to the EAU, and to evaluate the results in light of current guidelines.

## Material and methods

The local Data Protection Officer approved this retrospective study and issued a waiver from informed consent (18/13815).

**Fig. 1:**
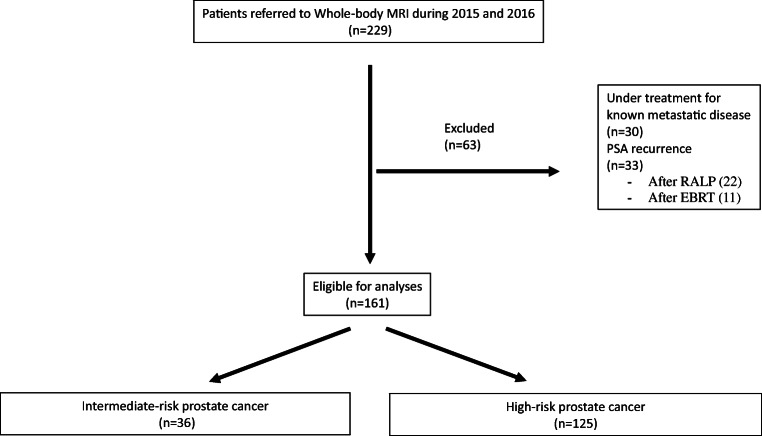
A flow-chart displaying all included and excluded patients in this study

Patient selection is shown in Fig. 1.

### Inclusion criteria

All patients had treatment-naïve prostate cancer and were eligible for a metastatic workup according to the current EAU guidelines with PSA > 20 ng/ml and/or DRE ≥ cT2c, and/or Gleason grade group ≥ 3, and/or bone symptoms [1]. All patients were examined during 2016 and 2017, a period where whole-body MRI was the routine examination for metastatic workup.

### Exclusion criteria

Patients with PSA recurrence after previous curative treatment and patients who were under treatment for previously detected bone metastases were excluded.

The diagnosis of prostate cancer was based on prostate biopsies performed using MRI-transrectal ultrasound (TRUS) soft image fusion targeted biopsies [11]. In patients unable to undergo a biopsy due to comorbidity, the diagnosis was based on clinical suspicion.

According to the EAU risk classification, included patients were classified as either intermediate-risk (Gleason grade group 2–3, and/or PSA 10–20 ng/ml, and cT≤2b) or high-risk (Gleason grade group ≥ 4, and/or PSA > 20 ng/ml, and/or cT ≥ 2c) [1].

### MRI examination

**Fig. 2:**
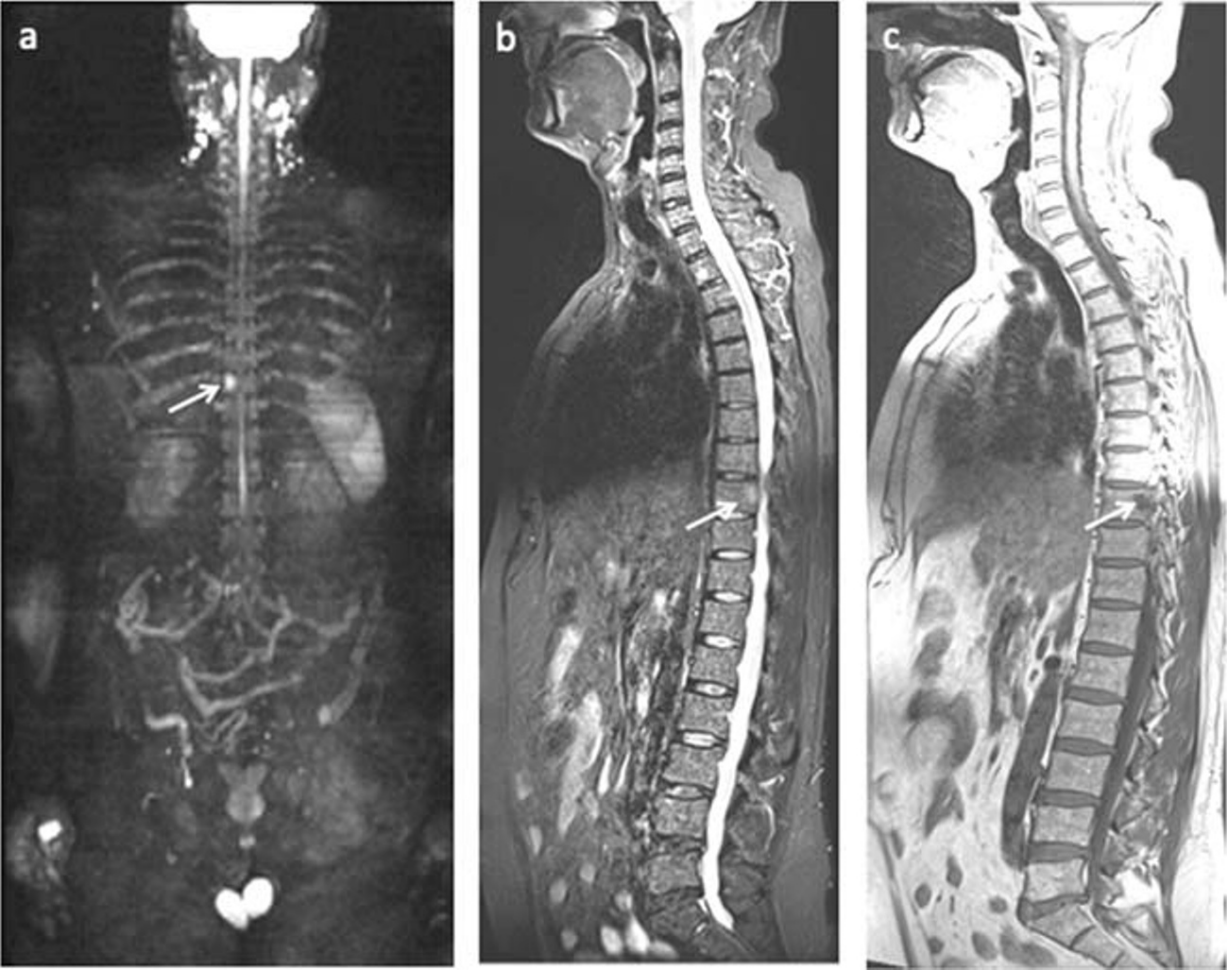
A 50-year-old patient with Gleason grade group 3 and PSA 23 ng/ml. **a** Coronal 3D MIP of b1000 s/mm^2^. **b** Sagittal STIR. **c** Sagittal T1w. The white arrows indicate a solitary metastasis in the 9th thoracic vertebra. A biopsy demonstrated chronic lymphatic leukemia

The MRI was performed on a 1.5-T Avanto (Siemens Healthcare), and included the following sequences: sagittal T1w of the spine and pelvis, axial diffusion-weighted imaging (DWI) using b1000 s/mm^2^ of the spine and pelvis, sagittal short tau inversion recovery (STIR) of the spine (Table 1). A 3D maximum intensity projection (MIP) was constructed from the b1000 s/mm^2^ images. Figure 2 demonstrates an example of whole-body MRI.

**Table 1:** MRI acquisition parameters

Sequence	Region	Plane of acquisition	Time of repetition (ms)	Time of echo (ms)	Slice thickness (mm)	Voxel size/reconstructed (mm × mm × mm)	Field of view (mm × mm)	Scan time (min:sec)
T1_tse	Pelvis	Transversal	483	7.9	4	1.5 × 0.91 × 4.0/0.9 × 0.9 × 4.0	350 × 350	5:01
T2_STIR	Spine	Sagittal	4000	71	3	1.95 × 1.56 × 3.0/1.56 × 1.56 × 3.0	400 × 400	2:54
T1_tse	Spine	Sagittal	400	10	3	1.39 × 1.04 × 3.0/1.04 × 1.04 × 3.0	400 × 400	2:36
DWI (ep2d_diff_b1000)	Skull base-thigh	Transversal	9000	69	5	3.79 × 3.79 × 5.0/1.89 × 1.89 × 5.0	500 × 424	3:09
T1_tse_mbh	Skull base-thigh	Transversal	596	8.7	5	1.86 × 1.30 × 5.0/1.30 × 1.30 × 5.0	500 × 500	1:18

*tse* turbo spin echo, *STIR* short tau inversion recovery, *DWI* diffusion-weighted images, *mbh* multiple breath-holds

### Image reporting

Imaging results were based on the original MRI reports performed by consulting radiologists specialized in oncological imaging. Twelve radiologists were involved, and the level of experience varied from 5 to 20 years. The definition of a metastasis was descriptive and at the discretion of the reporting radiologist. Thus, reporting did not follow a structured scheme, but described as metastases if showing a high signal on DWI and STIR, and a low or iso-signal on T1w compared with muscle tissue.

In case of metastases, the anatomical region was classified as either (i) pelvis including sacrum or (ii) spine (cervical-thoracal-lumbal). Metastases were not routinely biopsied, except for clinical suspicion of metastases of origins other than prostate.

### Statistical analysis

The definition of a metastasis was descriptive based on the original MRI reports. The results were dichotomized as bone metastasis positive or negative, and the anatomical region of metastases was registered. PSA in those with and without metastases was compared using the Mann-Whitney *U* test. For prediction of bone metastases, the areas under the curves (AUC) for PSA and Gleason grade group were calculated. A *p* value < 0.05 was considered statistically significant. All analyses were performed on MedCalc Statistical Software version 15.11.4 (MedCalc Software Ltd.) and SPSS Statistics for Mac, version 25 (IBM Corp.).

## Results

During 2016 and 2017, 229 patients with prostate cancer underwent whole-body MRI and 161 patients aged 71 years (IQR 64–76) and with median PSA 13.0 (IQR 8–28) were eligible for analysis (Fig. 1). The indications for whole-body MRI are shown in Table 2. Intermediate- and high-risk prostate cancer were found in 36 (22%) and 125 (78%), respectively. In four patients classified as high-risk disease, a confirmatory biopsy was not obtained due to comorbidity. The PSA in these patients ranged from 22 to 2000 ng/ml. The PSA levels according to the Gleason grade groups are shown in Table 3.

**Table 2:** Indications for whole-body MRI in intermediate- and high-risk prostate cancer

	Reason for referral	*n*
Intermediate-risk disease		
Gleason grade group 2	Symptoms (1)	1
	Bone lesions seen on CT (1)	1
	Unknown (3)	3
Gleason grade group 3	Gleason grade group ≥ 3	31
Total		36
High-risk disease		
Gleason grade group 1	cT3 disease	1
	PSA > 20 ng/ml	3
Gleason grade group 2	cT3 disease	8
	PSA > 20 ng/ml	12
Gleason grade group 3	Gleason grade group ≥ 3	7
Gleason grade group 4	Gleason grade group ≥ 3	55
Gleason grade group 5	Gleason grade group ≥ 3	35
Unknown Gleason grade*	PSA > 20 ng/ml	4
Total		125

*Four patients did not undergo prostate biopsies due to comorbidity

**Table 3:** The PSA levels according to Gleason grade groups

Gleason grade group	*n*	%	Median PSA (ng/ml), IQR	
1	4	2.5	23.5	11.7–24.8
2	25	15.5	17.0	9.3–24.5
3	38	23.6	11.0	6.8–18.3
4	55	34.2	13.0	7.8–35.0
5	35	21.7	20.0	9.0–33.0
Unknown*	4	2.5	52.0	27.0–167.8
Total	161	100	13.0	8.0–27.5

*Four patients did not undergo prostate biopsies due to comorbidity

*IQR* interquartile range

**Fig. 3:**
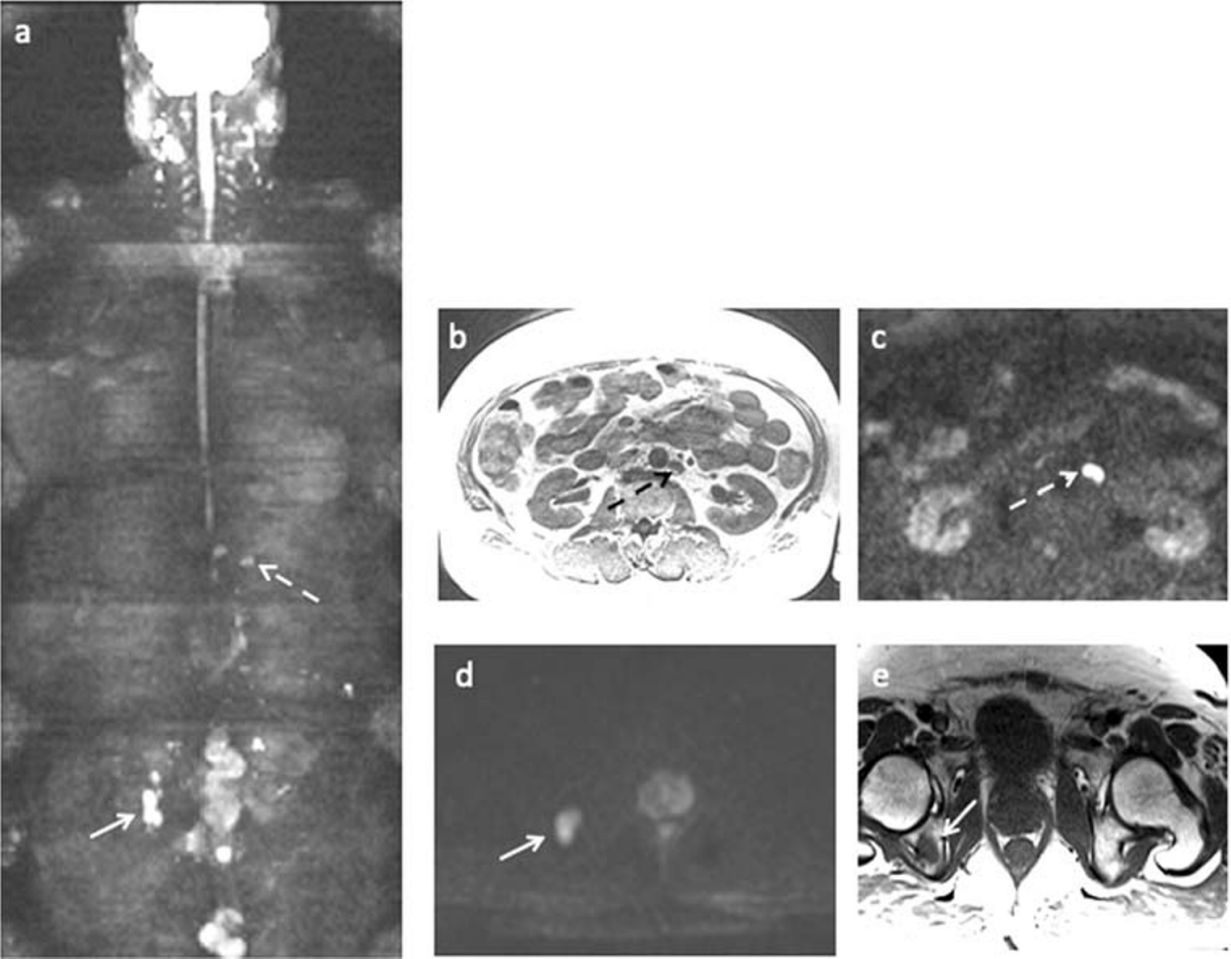
A 56-year-old patient with PSA 33 ng/ml and Gleason grade group 4. The white whole arrows indicate a bone metastasis in the right ischial tuberosity, while the stippled arrows indicate enlarged retroperitoneal lymph node. **a** Coronal 3D MIP based on DWI b1000 s/mm^2^. **b** Axial T1w. **c** Axial DWI b1000 s/mm^2^. **d** Axial DWI b1000 s/mm^2^. **e** axial T1w

Bone metastases were found in 8% (95% CI: 4–14, 13 out of 161), of which 23% (95% CI: 5–67, 3 out of 13) had less than four metastases. In one patient, a solitary metastasis was found in the 9th thoracic vertebra, but a biopsy from the lesion showed chronic lymphatic leukemia (Fig. 2). The remaining 7% (95% CI: 4–13, 12 out of 161) were treated clinically as if they had metastases from prostate cancer without a confirmatory biopsy. The prevalence of metastases in high-risk patients was 10% (95% CI: 5–17), and 83% (10 out of 12) were found in patients with Gleason grade group ≥ 4. The remaining metastases were found in two patients who did not undergo a confirmatory prostate biopsy. The pelvis was affected exclusively in 4 patients, and both the spine and pelvis were affected in the remaining 8. No patients demonstrated metastases in the spine without concomitant metastases in the pelvis (Fig. 3).

The median PSA in patients with and without metastases was 63.0 ng/ml (IQR 36.5–189.0) and 13.0 ng/ml (IQR 7.8–24. 5), respectively (*p* < 0.01). There was no difference in age (*p* = 0.3). The AUC for PSA and Gleason grade group for predicting bone metastases was 0.869 (95% CI: 0.75–0.99) and 0.762 (95% CI: 0.65–0.87), respectively.

## Discussion

This study demonstrated bone metastases in 7% of patients eligible for a metastatic workup defined by the updated EAU guidelines [1]. Since all metastases were found in EAU high-risk patients with Gleason grade group ≥ 4, our study suggests that a routine metastatic workup of intermediate-risk patients with Gleason grade group 3 might not be necessary.

In a recent study using whole-body MRI, Vargas et al found 1.5% bone metastases among 3765 patients with newly diagnosed prostate cancer [12]. However, that study also included low-risk patients, making comparison difficult. The prevalence of bone metastases is highly dependent on factors such as patient selection, method of imaging, previous treatment, PSA levels, and Gleason grade group in biopsy. All these issues complicate comparison of results.

The current guidelines are based on rather old studies using BS as imaging method [13–15], and BS is usually considered to be less accurate than both MRI and PET CT using choline- or prostate-specific membrane antigen [3, 16]. Previous BS studies typically report 3–30% bone metastases [13–15, 17, 18]. However, these studies may not be representative of current practice with widespread use of PSA measurements and MRI-guided biopsies. Therefore, new studies are needed for external validation of the guidelines, and definition of the optimal threshold for when to implement a metastatic workup with MRI.

The essential change in the updated guidelines is that a metastatic workup is now recommended for all patients with Gleason grade group ≥ 3 (Gleason score ≥ 7b), regardless of PSA and DRE. In our study, no metastases were seen in patients with Gleason grade group 3, while previous studies have demonstrated that a major Gleason grade pattern 4 is a significant predictor of a positive BS [13, 17]. The discrepancy compared with our study may at least in part be due to significant differences in PSA levels and possibly lower quality of the biopsies in earlier studies. We have previously reported 90% concordance between Gleason grade groups obtained by MRI-TRUS fusion targeted biopsies and prostatectomy specimen, while standard biopsies without MRI have demonstrated 50–75% concordance [11, 19, 20]*.* Therefore, it is possible that the Gleason grade group in biopsies may have been underestimated in studies prior to the MRI-guided biopsy era. Lastly, the false positive rate might be higher when using BS compared with that using MRI [3].

Unfortunately, we are unable to report cT-stage for the entire cohort, as cT-stage was often ambiguous or even absent from referrals and patient records. However, in nine cases, cT3 was the explicit reason for MRI referral (Table 2). This may reflect increasing reliance on imaging for staging among clinicians. We have previously shown that MRI is far more accurate than DRE for local staging [21]. However, the association between radiological T-stage and risk of metastases has to be determined before it may supplement or replace cT-stage in the guidelines.

In addition to the rather low prevalence of metastases found in our study, we did not find any metastases in the spine without concomitant metastases in the pelvis. This is in accordance with the findings by Woo et al who reported only one case of metastases exclusively outside the lumbar-pelvic region in a cohort of 308 patients with newly diagnosed prostate cancer [22]. In 1990, Cuming et al reported that only 2 out of 55 patients (3.6%) with a positive BS had metastases in the spine without involvement of the pelvis or lumbar spine [23]. These findings suggest that an imaging protocol could be limited to the pelvis, and only in case of pelvic metastases, a supplementary examination of the spine would be performed. Such strategy would reduce scanning time for the majority of patients without reducing diagnostic accuracy.

In the current study, we used a combination of T1w, STIR, and DWI. We did not attempt to assess the performance of the different sequences, nor the optimal combination. We only used one high *b*-value (b1000 s/mm^2^) since our focus was detection, and high *b*-value images are considered sufficient for detection of malignant lesions [24]. T1w and STIR are among the most widely used MRI sequences for assessing bone metastases, but over the last years, many have added DWI to the protocol. Larbi et al recently demonstrated that T1+DWI and T1+STIR performed equal to T1+DWI+STIR [24]. This indicates that one could omit either STIR or DWI. Nowadays, Dixon imaging has gained tremendous interest as it can replace both STIR and T1w images. The result is a much faster image acquisition with similar diagnostic accuracy [25, 26].

Limitations of this study are the few cases of metastases, descriptive reference standard, and retrospective design. Neither the MRI acquisition, interpretation, nor reporting was according to the MET-RADS standards [27]. We performed a single value of b1000 s/mm^2^ DWI without apparent diffusion coefficient map, which may mask sclerotic metastases. However, this was compensated for by adding T1 and STIR. We cannot report cT-stage for the entire cohort, and we did not have a systematic follow-up program in case of negative results. Lastly, we did not perform BS or CT for comparison with MRI. The STAMPEDE study showed that radiotherapy of the prostate in addition to hormonal therapy improved survival in patients with a low metastatic burden, e.g., less than four bone metastases on BS or CT [28]. However, a shift from conventional BS to MRI would most likely result in earlier and higher number of detected metastases. The impact of MRI upon treatment choice and survival needs further investigation.

In conclusion, this study suggests that the overall rate of bone metastases using the EAU guidelines for screening is low. Since we did not find any metastases in patients with Gleason grade group 3, the need to screen these patients should be further investigated. Also, it may be sufficient to scan the pelvis only, and perform a complete examination of the spine if metastases are detected in the pelvis.

## Abbreviations

AUC: Area under the curve
BS: Bone scan
cT-stage: Clinical T-stage
DWI: Diffusion-weighted imaging
EAU: European Association of Urology
ISUP: International Society of Urogenital Pathologists
MIP: Maximum intensity projection
MRI: Magnetic resonance imaging
PSA: Prostate specific antigen
STIR: Short tau inversion recovery
TRUS: Transrectal ultrasound
